# Functional profile and encapsulating properties of Colocasia esculenta (Taro)

**DOI:** 10.1002/fsn3.3357

**Published:** 2023-04-05

**Authors:** Muhammad Waqas Zubair, Ali Imran, Fakhar Islam, Muhammad Afzaal, Farhan Saeed, Syeda Mahvish Zahra, Muhammad Nadeem Akhtar, Muhammad Noman, Huda Ateeq, Muhammad Arslan Aslam, Shilpa Mehta, Mohd Asif Shah, Chinaza Godswill Awuchi

**Affiliations:** ^1^ Department of Food Sciences Government College University Faisalabad Pakistan; ^2^ Department of Environmental Design, Health and Nutritional Sciences Allama Iqbal Open University Islamabad Pakistan; ^3^ Institute of Food Science and Nutrition, University of Sargodha Sargodha Pakistan; ^4^ University Institute of Diet and Nutritional Sciences The University of Lahore Lahore Pakistan; ^5^ Department of Electrical and Electronic Engineering Auckland University of Technology Auckland New Zealand; ^6^ Adjunct Faculty, University Center for Research and Development, Chandigarh University Gharuan Mohali Punjab India; ^7^ School of Natural and Applied Sciences Kampala International University Box 20000 Kansanga Kampala Uganda

**Keywords:** *Colocasia esculenta*, functional properties, industrial application, probiotics

## Abstract

Especially in tropical and subtropical countries, tuber and root crops have developed into important food crops. Due to its use in food preparation, aesthetics, and medicine, taro (*Colocasia esculenta*) is ranked as the fifth most important root crop. In comparison, it stores a considerable quantity of starch – even more than potatoes, sweet potatoes, cassava, and other similar crops. *Colocasia* leaves are lower in calories and high in dietary fiber minerals and proteins. The corms of *Colocasia antiquorum* contain anthocyanins such as pelargonidin‐3‐glucoside, cyanidin‐3‐glucoside, and cyanidin‐3‐chemnoside, which are reported to possess antifungal and antioxidative characteristics. The underground corms of taro (*Colocasia esculenta*), which contain 70%–80% starch, are the primary reason for its cultivation. Taro is a highly digestible root vegetable with a high content of mucilaginous gums and trivial starchy granules. It is used to make a variety of dishes. This review article highlights the functional properties, phytochemical profile, encapsulating properties, and various industrial applications. Its health advantages and dietary uses were also addressed.

## Graphical abstract

The corms of *Colocasia antiquorum* contain anthocyanins such aspelargonidin‐3‐glucoside, cyanidin‐3‐glucoside, and cyanidin‐3‐chemnoside, reported to possess anti‐inflammatory and anti‐oxidative characteristics. This study highlights the functional properties, physicochemical composition, encapsulating properties, industrial application, probiotic, as well as prebiotic potential of taro.

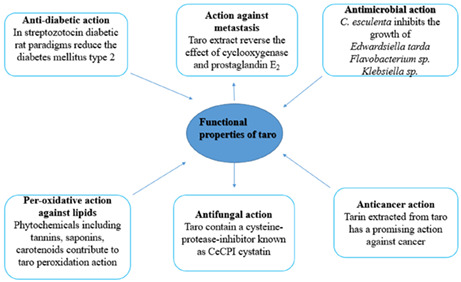

## 1 INTRODUCTION

Taro starch (70%–80%) is said to be the least expensive ingredient for the food industry because of its many possible uses in cuisine, such as a stabilizer, emulsifier, fat replacement, and filling agent. The excess glucose produced by photosynthesis is converted into starch, which serves as the store for plant's food source. In the presence of specific enzymes and water that are pulled from the cell to nourish the plant tissues, starch splits into its monosaccharide units (glucose) as needed (Britannica, 2021). Granules of starch are used to store starch in chloroplasts and in storing structures such as the roots of cassava, sago stem pith, maize, potato tubers, wheat, and rice seed. Plant starch is disassembled into its component sugar units in humans and other animals, which subsequently provide energy to the tissues (Dereje, 2021). Taro is a member of the family Arecaceae and the genus *Colocasia*. It is frequently grown for its subterranean corms all over the world (Melese et al., 2015). *Colocasia* plants are edible arums with enormous leaves and one or more starchy food‐storing stems (corms) in their subterranean stems (Adane et al., 2013). Taro starches comprise protein (5.6%), ash (0.8%), and phosphorus (0.4%), but a small lipid concentration in contrast to certain other tropical roots such as tiger nut and sweet potato (0.3%). Lower amylose concentration, high swelling capacity, good stability for water and oil retention, and additional useful properties of taro starch make it a popular choice with a bright future in the food processing industry. Small granular size (1–5 m diameter) and higher digestibility of taro starch suggested its high potential for creating functional foods like infant formula, bread, noodles, filler ingredients in edible coatings (Singla et al., 2020). Starches are becoming increasingly popular in various nonfood and food uses, owing to their low cost, ease of availability, biodegradability, and neutral behavior (Shashi & Dhull, 2020). Granules of native starch cannot dissolve in water, are inert, difficult to hydrolyze by enzymes, unable to sustain changes in temperature, pH, or shear stress due to retrogradation and linked syneresis, limiting their use in industrial applications (Punia, 2020). The current review focuses on the physicochemical composition, functional properties, phytochemical profile, prebiotic potential, probiotic encapsulation, and industrial applications of taro starch.

## 2 TARO STARCH

Root crops and tubers are extensively grown as the main crops, mainly in subtropical and tropical regions (Awuchi, 2023a). Among root crops, taro “*Colocasia esculenta*” is the fifth most significant crop growth owing to its ornamental, food formulation, and medicinal properties. In contrast to cassava, potatoes, sweet potato, and other starches, it contains a significant concentration of starch (Chao et al., 2020; Rashmi et al., 2018; Singla et al., 2020). Because of its multidimensional potential in food as an emulsifier, stabilizer, fat replacement, and filling ingredient, starch of taro corm (70%–80%) is regarded as a low‐cost choice for the food industry. Recently investigated outputs include baby meals, innovative packaging components, and geriatric meals including resistant starch (Agama‐Acevedo et al., 2011; Kaushal et al., 2015). Moreover, qualitative properties of taro starch enhance its developmental adaptableness following modification in a considerable reasonable manner than innate starch (Nagar et al., 2021; Whistler et al., 2012). “In vitro bile”: the ability of taro‐resistant starch to bind acids was identified as having promising health benefits owing to its ability to reduce cholesterol (Simsek & El, 2012). The starch found in the big corms of taro is easily digested, making it a rich source of glucose and, to a lesser extent, potassium and protein (Ahmed & Khan, 2013). Taro is mostly used as food, and its major components are corms and its edible leaf. Taro may therefore be transformed into nonperishable goods through food manufacturing procedures, improving their nutritional content, lengthening their shelf lives, and reducing food waste. Due to its high moisture, enduring metabolic activity, and microbial invasion, taro is particularly susceptible to postharvest losses, which limit its shelf life and induce harm during harvesting (Saxby et al., 2021). Individuals with peptic ulcers, pancreatic illness, chronic liver issues, gall bladder, and inflammatory bowel disorder can also benefit from taro starch. The tiny, fine granules of taro starch are readily absorbed, the starch is hypoallergenic, but it is gluten free (Rashmi et al., 2018; Simsek & El, 2012).

## 3 CHEMICAL COMPOSITION OF TARO STARCH

The nutritional profile of taro corm, similar to other root crops, is high in carbohydrates but lower in fat and protein Table 1. It has a high potassium content and a moderate phosphorus content. Taro corm is high in minerals, and the little starch granules aid in increasing the bioavailability of nutritional content through improved digestion and absorption (Das & Sit, 2021; Saxby, 2020). Taro has a higher protein content than other root crops, because the root and rhizome contain symbiotic soil microorganisms. These bacteria repair microorganisms in the air and raise nitrogen levels in the leaf and corm (Lucy et al., 2004). Furthermore, owing to the discharge of growth hormone to the root and distribution to the entire plant, bacteria are utilized as a plant growth enhancer. The ability of taro crop to develop in a variety of environmental and ecological situations is aided by the free‐living character of these soil bacteria (Lucy et al., 2004). These features are both economically and environmentally significant. Vitamin C and vitamin B complex (riboflavin, thiamin, and niacin), which are vital components of the human diet, are abundant in taro corms and leaves. Most other vitamins are lacking in roots and tubers, though they do contain considerable amounts of dietary fiber (Temesgen & Retta, 2015). Taro‐cooked leaf includes folic acid, iron, and beta carotene, all of which help to prevent anemia. One pound of malanga flour has roughly 1530 calories (Rincón‐Aguirre et al., 2018). Malanga flour has the following composition: 75.5%carbohydrates, 5.1% protein, 6.8% minerals 1.6% fat, 1.2% water, and 9.8% fiber (Hoyos‐Leyva et al., 2018).

**TABLE 1:** Chemical composition of taro croms.

Constituents	%	References
Starch	99.7	Zeng et al. (2014), Tattiyakul et al. (2007)
Moisture	11.5	
Ash	0.28	
Protein	0.35	
Amylose	19.2	
Crude fiber	0.94	

## 4 PHYTOCHEMICAL PROFILE OF TARO

### 4.1 Carotenoids

Among the most important groups of phytochemical substances is the carotenoids. They are referred to as tetraterpenoids because they have eight isoprene (C5) units and include the carbon atom C40. Distinct structural alterations to this particular structure, including hydrogenation, isomerization, cyclization, among others, can produce a variety of numerous carotenoid compounds. Due to the linking isoprene units, the connected double‐bond structure in carotenoids is what makes them most distinctive (Rodriguez, 2001). This system functions as a light‐absorbing chromophore that particularly absorbs red, orange, or yellow light at a certain wavelength. Yellow papaya and carrots, orange sweet potatoes, and red peppers are a few examples of these foods. Because of this, there are different carotenoid species found in different food types, which results in different food colorings. Carotenoids retain their lipophilic characteristics due to their structural makeup, which enables them to bond to lipid molecules. Although carotenoids are produced in plants from start, microbes may also produce them (Mendes‐Silva et al., 2021).

### 4.2 Polyphenolic components

Phytochemicals belong to the large family of phenolics. They are classified based on the number of phenolic units in their chemical makeup. The amount of phenolic rings, how they are arranged, and the various chemical components that are linked to such rings are used to describe any of these particular units (Awuchi, 2023a; Zeghoud et al., 2023). The following groups of substances can be further broken down: stilbenes, flavonoids, lignans, tannins, and phenolic acids (Pandey & Rizvi, 2009). Flavonoids all share a basic three‐ring composition with two benzene rings joined by a three‐carbon atoms to create an oxygenated heterocycle. As its categorization includes a greater variety of subclasses, including flavonols, flavan‐3‐ols (also known as flavanols), flavanones, flavones, isoflavonoids, and anthocyanidins, flavonoids make up a bigger majority of polyphenolic studies. The phenolics known as phenolic acids – also known as hydroxycinnamic acid and hydroxybenzoic acid – contain one carboxylic acid component (Kumar & Goel, 2019). Two phenyl units are joined by a two‐carbon–methylene bond to form stilbenes. Lignans not only have a 2,3‐dibenzylbutane composition, but also two phenolic units. Finally, tannins have the capacity to attach to protein structures because they include free total phenolic groups (Faye et al., 2021). Similarly, carotenoids where each form of polyphenolic molecule has a distinct biochemical structure that allows for variable light absorption, giving the crop a particular color. As a result, each polyphenolic component is tested at a particular wavelength using various solvents.

## 5 FUNCTIONAL PROPETIES OF TARO STARCH

### 5.1 Antimicrobial properties

Foodborne diseases are brought on by eating food that has been infected with toxic substances or pathogenic microorganisms (Awuchi, 2023b); Table 2. People all across the world are being affected by this growing health problem. The most typical signs of foodborne illnesses were stomach pain, vomiting, diarrhea, and nausea (Kadariya et al., 2014). Antibacterial activity of *C*. *esculenta* was attributed to its aqueous accumulation. *Edwardsiella tarda*, *Flavobacterium* sp., *Klebsiella* sp., *Vibrio cholerae*, *Aeromonas hydrophila*, *Escherichia coli*, *Salmonella* sp., *Vibrio parahaemolyticus*, *Vibrio alginolyticus*, and *Pseudomonas aeruginosa* were among the microorganisms studied. Several low‐fixation microscopic species and parasites were shown to have high antibacterial action in *C*. *esculenta* (Singh et al., 2012). Thakur & Modi (2020) looked into the antibacterial and proximate analyses of fermented taro skin that was purchased from a nearby manufacturer of Poi. One of the strains recovered from the material, *Leuconostoc mesenteroides*, was found to have antibiotic activity versus the examined bacterial strains. Poi was shown to encourage the growth of bacteria that produce bacteriocin (Muller et al., 2002). In research, the antibacterial effects of leaf and taro tuber against nine clinical infections were evaluated by Chakraborty et al. (2015). Antimicrobial action was assessed using the zone of inhibition that emerged during the incubation time. While leaf extract had the strongest efficacy against *Proteus mirabilis* at 100 mg/mL concentration, the largest inhibition zone for tuber extraction at that dose was recorded versus *Klebsiella* sp. It was discovered that tuber extract has more potent antibacterial properties than leaf extract (Chakraborty et al., 2015).

**TABLE 2:** Functional properties of taro.

Name	Study	Effect	Results	Reference
2% (w/v) Taro	Growth and adherence of *Lactobacillus* species (in vitro)	Improvement of human gut microbiota through prebiotic potential of taro	↑ growth of *L*. *acidophilus*, *L*. *paracasei*, and *L*. *plantarum* ↑ self‐agglutination of *L*. *paracasei*	Saxby et al. (2019)
Aqueous extract of taro	*Streptococcus mutans*	Antimicrobial activity	↓ growth	Singh et al., (2012)
Ethanolic extract of taro	Alloxan‐induced diabetic mice	Antihyperglycemic effect	Reduced glycemic activity	Singh et al.(2012)
Extract of taro 400 mg/kg bw	Male Sprague Dawley rats	Antidiabetic and antianemic effect	Inhibited aldose reductase enzyme activity, increased production of hemoglobin	Sulistiani et al. (2020)
Ethanolic leaves concentrate	Wister rats, granuloma model	Anti‐inflammatory activity	Calmed the inflammation	Keerthy and Joshi (2019)
Ethanolic and aqueous extract produced five digalactosyl‐diacylglycerols (DGDG) and three monogalactosyl‐diacylglycerols (MGDG)	Human Caco cell lines	Anticholesterol synthesis, declined cholesterol‐induced colorectal cancer (CRC)	Inhibition of human lanosterol synthase (hOSC), declining risk of CRC	
Ethanolic taro leaf extracts 10–50 mg/mL	Earth worms	In vitro anthelmintic activity	Potent paralysis and death time	Kubde et al. (2010)
25% Alcoholic concentration of taro leaf extract	*Alternaria ricini* and *Alternaria solani*	In vitro antifungal activity	Inhibition of pathogenic growth	Mengane (2015)
Taro leaf juice	In vitro rat liver slice model	Hepatotoxins (CCl_4_ and acetaminophen) induced lipid peroxidative reactions	Prevented the elicit of these reactions and protected liver	Patil and Ageely (2011)

### 5.2 Antidiabetic effects

Among the most difficult diseases of the 21st century, diabetes causes vital biochemical processes in the body (metabolism of carbohydrates, proteins, and lipids), and its incidence is increasing worldwide, especially in rural Nigerian people (Ani et al., 2011). Alternative approaches are desperately needed since contemporary therapy is unable to manage all of the pathophysiological features of the condition and because doing so has a huge negative impact on the economies of the world's emerging nations. Rural people's life may be significantly impacted by the use of medicinal plants in the conventional management of diabetes mellitus, particularly in remote regions of developing nations with limited access to healthcare services. Diabetes has reached epidemic proportions, with more than 350 million persons likely to be affected by 2035 (Gheith et al., 2016). According to the study, there may be broken progress in the search for plants that may stop the progression of diabetic nephropathy with the use of cocoyam and immature plantain flour in the diet treatment of type 2 diabetes mellitus reported by Eleazu et al. (2013).

### 5.3 Anticancer activities

Cancer is the greatest cause of mortality in the world, and it is mostly caused by poor eating habits and a sedentary lifestyle. It is critical to discover strategies to lower and prevent cancer risk through dietary components found in plant foods. Cancer is a multistage illness, and tapping at any stage might assist reduce the severity of the disease. Phytochemicals from the roots and tubers have been shown to have anticancer properties in a variety of carcinoma cell lines and animal models (Rashmi et al., 2018). In vitro actions of chemicals produced from taro on carcinoma cells of colon were initially described by Brown et al. (2005). Of 130 plants examined, Sakano et al. (2005) found that only taro reduced the human enzyme that produces cholesterol–lanosterol synthase (hOSC). Because excessive blood cholesterol concentrations have been associated with an enhanced incidence of colonic carcinoma and CRC, inhibiting triglycerides synthesis can assist to lower cholesterol formation (Wang et al., 2017). Additional study is necessary, but tarin extraction from taro has promising action as a possible cancer therapy (Pereira et al., 2018). Even yet, more in clinical and in vivo research are required to fully understand the significance of tarin as a sustainable origin for preventing cancer spread, colonization, and migration.

### 5.4 Peroxidative activity against lipids

Numerous phytochemicals included in taro could prevent peroxidation. Numerous substances including vitamins, tannins, carotenoids, alkaloids, flavonoids, and saponins are thought to contribute to antioxidant qualities of taro (Aja, Chiadikaobi, et al., 2023; Aja, Ogwoni, et al., 2023; Eleazu et al., 2016). Entire leaf juice of *C*. *esculenta* was used to account for the free radical rummaging feature. The effect of in vitro free radical rummaging on liver cells was investigated using animal liver cut models. Brooding the liver cuts around the cytotoxic centralizations of acetaminophen and carbon tetrachloride (CCl_4_).

### 5.5 Activity against metastasis

The most common cause of breast malignant growth death is a case of metastatic infection. Both hypothetically and directly, the compounds derived from the fundamental foundations of the *C*. *esculenta* plant can inhibit tumor spread. It showed evident mobility in a preclinical breast carcinoma model. Taro reverses the effects of a comparable suppressed combination of mRNA for cyclooxygenase 1 and 2 and prostaglandin E_2_ (PGE_2_) directed downward. Taro extract substantially stops the proliferation of a certain, though not all, breast and testicular cancerous cell lines, and it entirely blocks the movement of tumor cells (Kundu et al., 2021).

### 5.6 Antifungal properties

Yang and Yeh (2005) used recombinant efficiency articulation and atomic cloning to investigate the antifungal motion of taro a cysteine–protease inhibitor known as CeCPI cystatin, discovered in the taro corm *C*. *esculenta*. The assay revealed that the recombinant CeCPI protein showed unmistakable cysteine–protease inhibitor movement. As a result, the study discovered that the plant has a basic toxic impact on phytopathogenic parasites' mycelium formation (Yang & Yeh, 2005).

### 5.7 Prebiotic potential of taro starch

**FIGURE 1:**
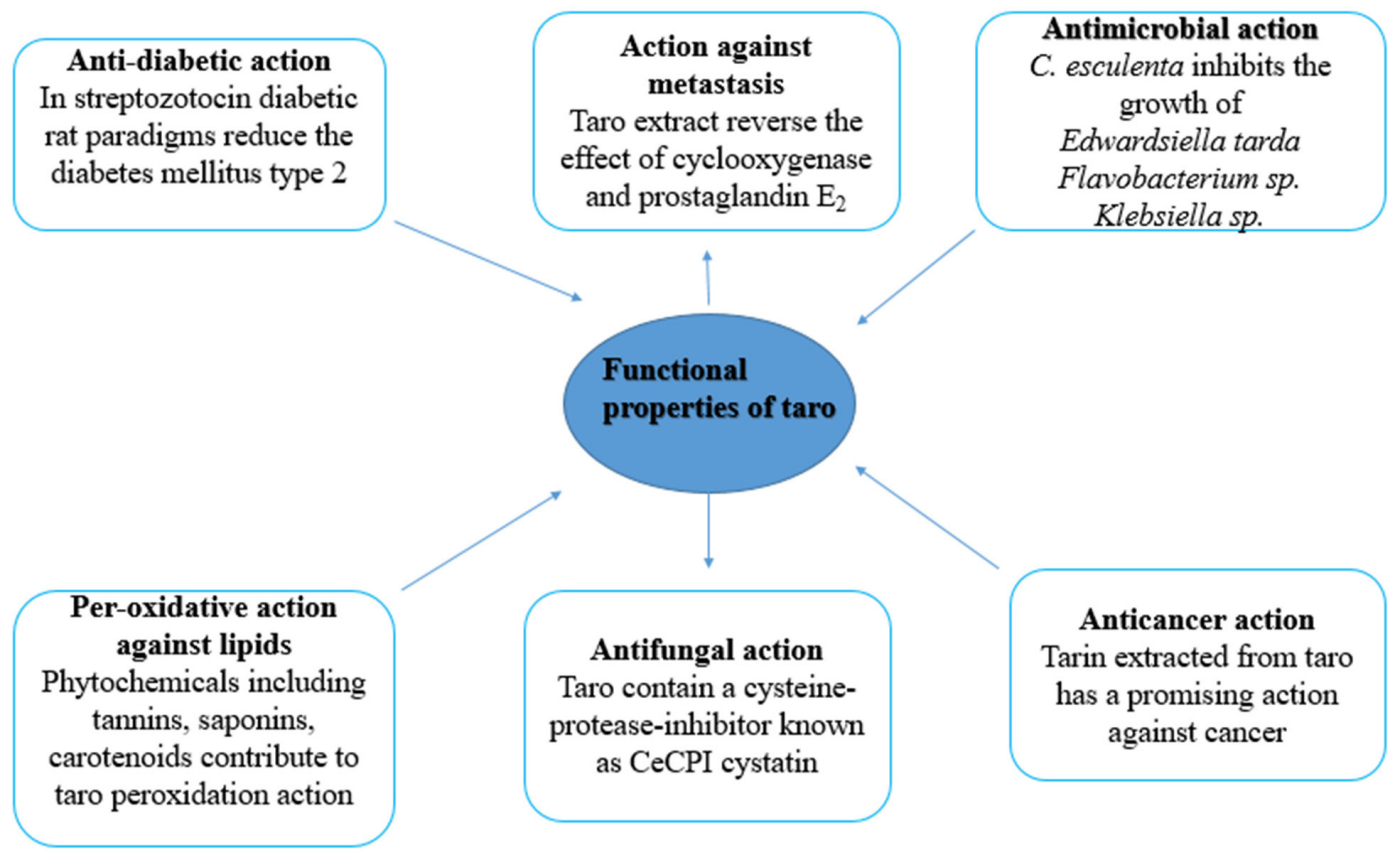
Depicts the functional and encapsulating properties of taro starch.

Dietary fibers include water‐soluble nonstarch polysaccharides that has been linked to improved gut health and immunological function (Sanders et al., 2019). The potential of WS‐NSPs to mitigate digestion, function as a source of nutrients for useful gut bacteria, and attach to intestinal epithelium (IECs) in the digestive system (GI) is credited with the positive effects gained from their usage (Schellack & Combrinck, 2020). There is currently no information on Tc digestibility, cytotoxicity of WS‐NSP in the HT‐29 cell, prebiotic prospect, or ability to control the production of cytokines, especially IL‐8, in HT‐29 cells. In this investigation, the gut epithelial cell HT‐29 was employed as it is a well‐known and commonly used in vitro model for examining enterocyte–immune responses, namely IL‐8 production reported by Anwar et al. (2021). WS‐NSPs are known for their potential to enhance the growth of probiotic strains, which gives them a prebiotic effect. The prebiotic ability of WS‐NSPs is largely due to their ability to withstand digestion and develop into an easily fermentable form of carbohydrates for advantageous microbes in the digestive system (Bindels et al., 2015; Figure 1).

## 6 APPLICATION OF TARO STARCH IN FOOD INDUSTRY

The look, flavor, and acceptance of bread in general made from an unbalanced blend of wheat and taro starch received the highest ratings from the panelists. Owing to the occurrence of a briny taste and a strange flavor in the blended bread, the acceptability of taro–wheat bread diminished as the taro flour blending ratio increased. GFB samples had a higher resistant starch content than WFB samples. As the amount of taro flour added to the diet increased, the dietary fiber content increased significantly (Abera et al., 2017; Ammar et al., 2009; Arıcı et al., 2020; Emmanuel et al., 2010). Taro starch had a starch content of 67.7%, 23.8% amylose content, 14.8% swelling power, and 21.9% solubility. The sensory evaluation revealed that the majority of panelists enjoy cookies produced with modified taro starch (Yesi & Sugiarti, 2021).

## 7 PROBIOTICS

The term “probiotic” is not limited to typical probiotics in the definition. Candidate probiotics will undoubtedly be extracted from novel sources, with previously unforeseen activities and interesting, new health advantages (Afzaal et al., 2022). This definition does not exclude so‐called subsequent generation probiotics, which can be thought of as live biotherapeutics in some situations. Nevertheless, based on the stated usage, proper safety, ethical, and legal issues must be talked in the production of probiotics like these adhering to whenever appropriate, the Nagoya Protocol and obtaining appropriate informed consent when isolating microorganisms from humans (Johansen, 2017). Correct strain identification and naming are critical components of appropriate probiotic characterization. Probiotic strains should be labeled with the most up‐to‐date bacterial nomenclature, as defined by the International Nomenclature Code (Binda et al., 2020). The website http://www.bacterio.net/ has an up‐to‐date list of prokaryotic names with nomenclature standing (Parte, 2018). Regardless of previous research, there is a necessity to specify these criteria accurately and clearly, as well as provide insights into how to meet them, without getting into the minutiae of probable mechanical requirements. Probiotic strains must, in brief, (1) be appropriately categorized, (2) harmless for the desired use, (3) endorsed by at least one successful human clinical study carried out in accordance with broadly agreed scientific standards, and (4) alive in adequate figures in the commodity at an effective dose throughout life span (Jackson et al., 2019). The usage of probiotics is widely recognized as a natural way to support good health in both humans and animals. The features of a specific probiotics strain are intimately linked to the probiotics' mode of action. Selection is a difficult task that necessitates a foundation of basic information about a candidate strain's physiology and genetics as it relates to their intestinal function, therapeutical activities, and interactions with other microflora (Flach et al., 2018). When considering the origin of strain, growth, and genetics features in vitro and in vivo, it is critical to conduct a thorough investigation into its origin, genetic makeup, and growth characteristics. Probiotics must be able to exert a positive consequence on a host, resist transits through GI tract, stick to the intestinal epithelium cell membrane and populate the tract lumen, generate antimicrobial compounds versus microorganisms, be technologically acceptable for industrial operations, and be associated with clear health advantages. Specifically, in the case of new microbes and genetically modified strains like probiotics, it is necessary to consider their safety and danger while developing new foods (Binda et al., 2020; Shewale et al., 2014).

### 7.1 Application of probiotic bacteria in food industry

Probiotic bacteria are commonly encapsulated to improve viability during processing and for targeted distribution throughout the gastrointestinal tract. Probiotics are found in fermented dairy products, pharmaceuticals, and nutritional supplements. They play an important part in human health. The capacity of the microorganisms to endure in the human gastrointestinal system is debatable. Whey protein isolate alginate, gelatin, chitosan, and derivatives of cellulose are a few examples of biopolymers utilized for encapsulation to protect the sustainability of probiotic bacteria, and several encapsulation processes, such as extrusion, spray drying, and emulsion, have been documented (Huq et al., 2013; Morya et al., 2022). Since 2007, lists of species believed to be harmless for human use in foods have been maintained by the European Food Safety Authority (EFSA). “Qualified Presumption of Safety” (QPS) concept, goes beyond history of safe usage (EFSA, 2007). The QPS method is a proof‐based, detailed, and frequently updated method of communicating about the safety of certain microorganism species (Brodmann et al., 2017). The list is a good starting point for determining the safety of food strains that belong to a QPS species, as long as the strain‐specific testing mentioned next is done as well. The list is based on historical facts, frequent examination of the knowledge base, and rigorous scientific literature reviews, and it applies to a wide range of microbes present in the food chain (EFSA, 2020). The addition of the probiotic DS results in a doubling of total cholesterol decrease in the test group compared to the control group, indicating that DS is highly effective. Compared to the animals in the control group, the use of probiotic DS is associated with a substantial decrease in triglyceride levels of 37% and 45% in the “linen” and “cedar” groups, respectively, against the backdrop of an atherogenic diet. Blood serum's high‐density lipoprotein concentration in experimental animals increased by 45% in the “linen” group and 40% in the “cedar” group, whereas low‐density lipoproteins reduced by 19% and 23%, correspondingly (Borges et al., 2018; Khamagaeva et al., 2021).

## 8 MICROENCAPSULATION

Microencapsulation is an alternate probiotic preservation strategy that provides a comfortable habitat for the encapsulated microorganisms, enhancing probiotic vitality. Encapsulation is defined as a mechanical/physiochemical strategy of shielding and segregating sensitive components, mostly of a bioactive type, from atmospheric variables such as pH, O_2_, light, and other factors that negatively impact the activity of bioactive composites. Bioactive composites can exist in any material. Covering/coating materials are the primary materials safeguarding these sensitive composites; polymers in nature prevent and control timely availability and play a role in maintaining its stability (Islam et al., 2023). To date, much research has been conducted on microencapsulation. Probiotic microencapsulation not only shields probiotic cells from the harsh outside environment, but also enables for controlled release of the cells in precise locations (Afzaal et al., 2023). Probiotic‐based functional foods are becoming increasingly popular. Yogurt is one of the most significant dairy‐based products for researchers because of its increased nutritional profile, particularly its benefits as a probiotic carrier (Afzaal et al., 2023). When bacterial cells multiply and create exopolysaccharides, encapsulation occurs spontaneously. The microbial cells are encased in their own secretions, which operate as a capsule or protective structure, decreasing material permeability through the capsule and thereby exposing them to harmful environmental influences. Exopolysaccharides are synthesized by many lactic acid bacteria, however, they do not create enough to completely enclose themselves (Shah, 2000). In the food industry, encapsulation can be used for a variety of purposes, such as the ability to conceal colors, flavors, or odors; the ability to provide a controlled release or sustained (both temporal and time‐controlled release); the ability to lengthen life span; and the ability to preserve components versus nutrient loss. A microcapsule is made up of a semipermeable, spherical, thin, and robust membrane that surrounds a liquid or solid core and has a width of a few microns to 1 mm. Encapsulation improves the survivability of bacteria by allowing for easier handling and dosage management. Various encapsulation processes are used to carboxymethyl cellulose (CMC), chitosan, alginate, gelatin, carrageenan, and pectin are a few examples of food‐grade polymers that can be encapsulated (Anal & Singh, 2007; Neamatshahi et al., 2012). Anthocyanins can be utilized in the food business to offer food a variety of hues as well as nutraceutical properties. It is, however, difficult to keep anthocyanins in their active condition in food products. The stability of anthocyanins is so low that they are sensitive to processing and storage conditions. Encapsulation is a good way to get these substances into food (Li et al., 2021). The increasing importance of functional foods in well‐being and human health has sparked interest in creating innovative probiotic and nutraceutical oral administration techniques. In food compositions, encapsulation might offer protection and long‐term delivery. The topic of probiotics encapsulation for use in both the nutraceutical and food industries is discussed. Nutraceuticals, probiotics, and encapsulation technologies principles and present market conditions, food applications, and microbial microencapsulation for use outside of the pharmaceutical business are among the subjects covered. Researchers looking at the stability and viability of encapsulated probiotics in food matrices are also acknowledged and addressed (Feng et al., 2020; Reque & Brandelli, 2021). Several techniques, such as the use of vitamins, are being investigated to increase probiotic tolerance to harsh environmental conditions (amino acids, peptides, and microencapsulation). Protecting probiotic living cells with a physical barrier against toxic surroundings is an approach that is gaining popularity.

## 9 CONCLUSION AND FUTURE TRENDS

Spherical aggregates of taro starch have been used as an enclosure material to serve in probiotics and bioactive compounds' microencapsulation. In developing countries, one of the least expensive sources of nutritional energy in form of carbohydrates is tubers. Taro has a greater variety of nutrients and vitamins as compared to other tubers in terms of nutrition. The corms of *C*. *antiquorum* contain anthocyanins, such as aspelargonidin‐3‐glucoside, cyanidin‐3‐glucoside, and cyanidin‐3‐chemnoside, reported to possess anti‐inflammatory and antioxidative characteristics. It has been explained how taro is used as a food constituent in canned goods, extruded paste goods, baby foods, and so on. Improvements to existing technologies, as well as additional value added, can make products more appealing to consumers. This article highlights the functional and encapsulating properties of taro and its industrial applications as well.

## FUNDING INFORMATION

All authors affirm that no financial assistance was received for this study.

## CONFLICT OF INTEREST STATEMENT

All authors have affirmed zero conflict of interest.

## Data Availability

Additional data will be made available on request.
